# Aripiprazole Improves Depressive Symptoms and Immunological Response to Antiretroviral Therapy in an HIV-Infected Subject with Resistant Depression

**DOI:** 10.1155/2010/836214

**Published:** 2010-03-30

**Authors:** Chiara Cecchelli, Giacomo Grassi, Stefano Pallanti

**Affiliations:** ^1^Department of Psychiatry, University of Florence, Florence, Italy; ^2^Institute of Neurosciences, Florence, Italy; ^3^Department of Psychiatry, Mount Sinai School of Medicine, New York, NY 10029, USA

## Abstract

Aripiprazole is the first medication approved by the FDA as an add-on treatment for MDD. The impact of aripiprazole on the response to HIV is unknown. The patient we report on was diagnosed HIV-positive in 1997 and has been treated with antiretroviral therapy since then. In 2008, we diagnosed resistant major depression, hypochondria, and panic disorder. On that occasion, blood tests showed a significantly reduced CD4 count and a positive viral load. We treated this patient with aripiprazole and citalopram. Mood, somatic symptoms, and occupational functioning progressively improved. The last blood examination showed an increase in the CD4 count and a negative viral load. On the basis of the present case study and the review of the literature concerning the effects of psychotropic agents on viral replication, we suggest that the use of aripiprazole in HIV-infected subjects warrants further research.

## 1. Introduction

In November 2007 the Food and Drug Administration (FDA) approved the use of aripiprazole as an adjunctive, or add-on, treatment to antidepressant therapy in adults with major depressive disorder (MDD). Aripiprazole is the first medication approved by the FDA as an add-on treatment for MDD [1], modulating the dopaminergic system. Unlike typical and atypical antipsychotic drugs which act as antagonists to the dopamine D2 receptor, aripiprazole has a novel mechanism of action, causing it to act as a partial agonist to the dopamine D2 receptor. Partial agonists have a lower intrinsic activity to receptors than full agonists, which allows them to act either as a functional agonist or a functional antagonist, depending on the surrounding levels of the naturally occurring neurotransmitter (full agonist). In the absence of a full agonist, partial agonists show functional agonist activity, binding to the receptor so as to produce a response. In the presence of a full agonist, partial agonists show functional antagonist activity, inasmuch as receptor binding reduces the response from the level of that seen with the full agonist [2]. 

Drugs active on the dopaminergic system, such as methamphetamine, have been claimed to exert an effect on HIV infectivity [3, 4]. Lento-viral replication seems to be enhanced in peripheral blood mononuclear cells partially through dopaminergic activity. Dopamine acts through dopamine receptors (DRs), and classically DRs have been studied on neurons. In addition, DR expression has been reported in several types of peripheral blood leukocytes, including T lymphocytes and monocytes. Dopamine receptors have been shown to modulate the immune function of T lymphocytes [5–7]. The data show clearly that, with an increasing dose of methamphetamine, the replication of Feline immunodeficiency virus (FIV) is induced [3, 4, 8]. Psychostimulants, such as cocaine and methamphetamine, exert addictive and reinforcing effects through the elevation of extracellular dopamine (DA) levels in the CNS. The impact of aripiprazole, a dopamine stabilizer, on the response to HIV in HIV-infected patients is unknown. 

We present a case of resistant depression, somatoform disorder, and panic disorder in an HIV-infected subject, treated with aripiprazole as augmentation therapy. In this case aripiprazole led, not only to significant improvement in depressive symptomatology, but also to an improvement in CD4 cell count and viral load.

## 2. Case Study

The case we wish to present involves a 37-year-old Caucasian man, by trade a musician. Since adolescence, he has been beset by mood instability (cyclotimia), irritability, severe onicophagy, and obsessive-compulsive and impulsive personality traits, and hypochondria. He was in good health until 1992 when he was confirmed as having a gastric ulcer and put on omeprazole and prokinetics for several years. He was diagnosed HIV-positive in 1997 and has been treated with antiretroviral drugs since then (nevirapine and the association of lamivudine and zidovudine). In spring 2008 he began to manifest acute episodes characterized by shivers, nausea, retching, diarrhea, tremor, and paresthesias. He also complained of cephalea, cervical and lumbar pain, middle and late insomnia, decrease in appetite, fatigue, impairment in occupational functioning, diminished ability to concentrate and recurrent concerns about his health. Because of this symptomatology, he sought the advice of specialists in different fields (infective diseases, gastroenterology, neurology, orthopedics), who prescribed several diagnostic tests (blood tests, coproculture, X-ray of digestive tract, abdominal CT, and cervical rachis MRN). Blood tests showed a CD4 count of 200 cells/*μ*L and a viral load of 3000 copies/mL. All the other diagnostic tests were negative. In the meantime, he was prescribed different therapies to control anxiety, somatisation, and mood instability: prokinetics (domperidone 30 mg/die), omeprazole (20 mg/die), levosulpiride (50 mg/die), lorazepam (3 mg/die), trimipramine (100 mg/die) for 8 weeks, paroxetine (20 mg/die) for 10 weeks, and mirtazapine (30 mg/die) for 12 weeks. None of these drugs led to substantial clinical improvement. In September 2008 he came to our outpatient clinic owing to persistent symptomatology. We diagnosed resistant major depression, somatoform disorder (hypochondria), and panic disorder. Resistant depression was defined as a depressive episode with at least two previous failed antidepressant trials, each lasting at least 6 weeks (stage II of antidepressant resistance according to Thase and Rush) [9]. In concert with a specialist in infective diseases, we decided to treat his psychiatric symptomatology in order to improve his compliance with antiretroviral therapy. In our experience low doses of aripiprazole (up to 15 mg/die) are effective as augmentation therapy in depressed patients who are nonresponders to SSRI or tricyclic antidepressants in monotherapy; so we chose to use this drug for this patient, added on to citalopram 40 mg/die. For states of anxiety, we avoided benzodiazepines because of the patient's tendency to abuse it. So we used clonazepam 0.5 mg/die only at the beginning of therapy. Gradually, the frequency of the acute episodes decreased and the intensity of symptomatology during these episodes diminished. Mood, somatic symptoms, and occupational functioning progressively improved. The last blood examination showed up an increase in CD4 count (400 cells/*μ*L) and a negative viral load.

## 3. Discussion

Mental disorders are highly prevalent in HIV-infected individuals, with about 48% of these patients having a psychiatric disorder [10]. In HIV-infected patients, mental disorders such as depression have been associated with increased risk of poor medication adherence and HIV disease progression [11]. Moreover, patients who are depressed or suffer from anxiety or have substance abuse disorder at the time of initiating antiretroviral therapy have poorer virological responses to treatment. Improvement in depression after treatment has been shown to improve quality of life and increase in treatment adherence. 

The availability of highly active antiretroviral therapies (HAARTs) has not eliminated HIV-1 infection of the central nervous system (CNS) or the occurrence of HIV-associated neurological problems, which are both increasing due to the longer lifespan of infected individuals on antiretroviral therapies [12] and the poor ability of most antiretroviral drugs to penetrate the blood-brain barrier [13, 14]. Human immunodeficiency virus (HIV) enters the central nervous system (CNS) soon after initial infection [15] resulting in ongoing inflammation, as well as neurological damage that leads to the development of HIV-associated neurocognitive disorders (HANDs) in as many as 50% of infected individuals [16, 17]. HIV is thought to enter the brain through the transmigration of infected monocytes across the blood-brain barrier. Within the CNS, macrophages are the primary source of HIV and the virus spreads primarily through the infection of brain macrophages and microglia [18]. Infected macrophages produce numerous factors that are neurotoxic and contribute to the neurological damage that occurs in HIV-infected individuals [19]. 

The effects of aripiprazole, a dopamine stabilizer, on depressive symptoms are well documented [20], but its effects on viral replication and immunologic system call for further study. 

In 2000 Kristiansen and Hansen found that some psychotropic agents (flupentixol, paroxetine, femoxetine) and their structural isomers inhibit HIV replication in HIV-infected patients with AIDS related dementia [21], suggesting the use of these psychotropic drugs in combination with antiretroviral therapy to control the condition. Earlier investigations have shown that microorganisms with high affinity to the nervous system are very sensitive to neurotropic drugs [22–24]. Aripiprazole acts as a partial agonist at dopamine D2, D3, and at serotonin 5-HT1A receptors and as an antagonist at 5-HT2A receptors, so it should be interesting to test its antiviral activity.

As regards aripiprazole's immunological impact, we know that several antidepressants (tricyclic, IMAO, SSRI, and bupropion) show up such an effect in that they exert an immunomodulatory effect, reducing proinflammatory cytokines and increasing anti-inflammatory cytokines [25]. Antidepressants seem to exert their immunological effect both at central (CNS) and peripheral level (on activated macrophages). Aripiprazole might also be effective in HIV-infected patients for a specific immunological effect. Neuropsychiatric disorders, in HIV-infected patients, seem to occur, in part, through glial activation and release of cytotoxic chemokines and cytokines such as interferon-*γ*(IFN-*γ*) [26]. In vitro, aripiprazole has a significant inhibitory effect on IFN-*γ*-induced microglial activation, reducing the generation of NO and TNF-*α* and suppressing the elevation of [Ca^2+^] in microglia [27]. We suppose that aripiprazole has the same effect in vivo on astrocytes activated by infection.

Another possible reason for the improvement in the patient's immune status is an indirect effect of aripiprazole on cortisol levels brought about by improvement in depression. The dysregulation of the HPA axis function (e.g., increase in adrenocorticotrophin-releasing hormone and cortisol levels) has been associated with stress and depression in humans, and such dysregulation may negatively impact the immune response. There are data supporting the fact that glucocorticoids may affect HIV pathogenesis directly, through increased viral replication, or more indirectly by inhibiting the immune response to other pathogens. In fact, in 1997 Christeff et al. [28] demonstrated that high levels of plasma cortisol are negatively associated with CD4 cell counts. Thus, aripiprazole's effect on HIV replication and CD4 counts may be partially explained by an indirect effect on immune response brought on by decreased cortisol levels, in turn induced by improvement in depressive symptoms. 

As a final point, we cannot exclude the possibility that the improvement in depression symptomology may result in an increased adherence to antiretroviral treatment.

On the basis of the present case study and the review of the literature about the effects of psychotropic agents on viral replication, we suggest that the use of aripiprazole in HIV-infected subjects warrants further research. 
